# The pupil response reveals increased listening effort when it is difficult to focus attention

**DOI:** 10.1016/j.heares.2015.02.004

**Published:** 2015-02-27

**Authors:** Thomas Koelewijn, Hilde de Kluiver, Barbara G. Shinn-Cunningham, Adriana A. Zekveld, Sophia E. Kramer

**Affiliations:** a Section Ear & Hearing, Department of Otolaryngology-Head and Neck Surgery and EMGO Institute for Health and Care Research, VU University Medical Center, Amsterdam, The Netherlands; b Department of Biomedical Engineering, Center for Computational Neuroscience and Neural Technology, Boston University, Boston, USA; c Linnaeus Centre HEAD, Department of Behavioral Sciences and Learning, Linköping University, Linköping, Sweden

## Abstract

Recent studies have shown that prior knowledge about where, when, and who is going to talk improves speech intelligibility. How related attentional processes affect cognitive processing load has not been investigated yet. In the current study, three experiments investigated how the pupil dilation response is affected by prior knowledge of target speech location, target speech onset, and who is going to talk. A total of 56 young adults with normal hearing participated. They had to reproduce a target sentence presented to one ear while ignoring a distracting sentence simultaneously presented to the other ear. The two sentences were independently masked by fluctuating noise. Target location (left or right ear), speech onset, and talker variability were manipulated in separate experiments by keeping these features either fixed during an entire block or randomized over trials. Pupil responses were recorded during listening and performance was scored after recall. The results showed an improvement in performance when the location of the target speech was fixed instead of randomized. Additionally, location uncertainty increased the pupil dilation response, which suggests that prior knowledge of location reduces cognitive load. Interestingly, the observed pupil responses for each condition were consistent with subjective reports of listening effort. We conclude that communicating in a dynamic environment like a cocktail party (where participants in competing conversations move unpredictably) requires substantial listening effort because of the demands placed on attentional processes.

## 1. Introduction

Having a conversation with a good friend at a party can be relatively easy if you know where and when he or she is going to talk to you (e.g., Kitterick et al., 2010). On the other hand, talking at the same party with a group of people whom you do not know well and who are dancing or moving around feels much more effortful. Although multiple studies show that prior knowledge about where, when, and who is talking has a positive effect on speech recall performance (e.g., Ihlefeld and Shinn-Cunningham, 2008; Kitterick et al., 2010), there is little evidence that this information affects cognitive load during speech processing.

We showed in a previous study (Koelewijn et al., 2014) that dividing attention over two streams of information instead of focusing on one increases cognitive load. According to the ‘load theory of selective attention’ (Lavie et al., 2004), high cognitive load decreases performance, an effect observed in our study and in prior research (Best et al., 2010). We concluded that the amount of allocated attentional recourses affects cognitive load. If these attentional resources are deployed effectively, this should lead to better segregation of target information from background information and thus better performance (Broadbent, 1958). Effective early filtering should ease later semantic processing by reducing the amount of conflicting information vying for resources (Rönnberg et al., 2013), thereby reducing the total cognitive load. This was not addressed in our previous study (Koelewijn et al., 2014), where we only investigated the amount of cognitive resources needed to process two streams of information compared to one and not how effectively attentional processes could use available cues to facilitate target-masker segregation processes. For effective early filtering, listeners must be able to access relevant, salient cues that distinguish target from masker to enable attention to be properly focused on the target. In the current study, we investigate how the features location, speech onset, and voice (and other speech characteristics) of a talker affect speech intelligibility and listening effort.

Kidd et al. (2005) showed that in a complex listening task when there were two distractor talkers, prior knowledge about *where* the target speech is presented has a positive effect on performance. This effect was replicated by Kitterick et al. (2010), who simulated a complex listening environment in order to create challenges like those that arise at a cocktail party. The effects of uncertainty of speech location, speech onset, and target talker on speech perception were investigated by determining the benefits of constraining these three parameters during speech reception threshold (SRT) tasks. Target phrases were masked by at least 12 distracting phrases within each trial. Constraining where the target talker was located yielded a modest benefit of 1.0 dB in SRT when the target phrases and the masking phrases had different onset times relative to one another. When one of the masking phrases had an onset time similar to that of the target phrase, the benefit of location information reached 5.1 dB. In other words, the location information became more relevant when a distracting sound was presented at the same time. In a study by Best et al. (2007), visually guided attention was directed towards the location of a talker or a particular birdsong. Their results showed that knowledge about where a target is located improves its identification when presented with similar distractors.

The effect on speech perception of knowing *when* someone is going to speak has not been studied much. Best et al. (2007) showed that visually cueing the target onset had little effect on the ability to attend to and recall a spoken digit stream and a modest effect for birdsongs. Gatehouse and Akeroyd (2008) showed a small performance benefit for hearing-impaired listeners when the onset of a word was preceded by a visual cue. Kitterick et al. (2010) also showed a small effect of making speech target onset times more predictable. Thus, providing temporal information yields small benefits for the behavioral ability to attend to and recall speech.

Finally, Kitterick et al. (2010) showed that constraining *who* is going to talk affects speech intelligibility. In their study, the target talker was either fixed or randomly selected from of a group of talkers. When the same talker uttered several target phrases, participants were able to perform the task under less favorable listening conditions (lower signal-to-noise ratios, SNRs) than when the target phrases were uttered randomly by one of the talkers. The results suggest that prior knowledge about who is going to talk benefits speech processing. This is in line with the idea that familiar voices are more intelligible than novel voices (e.g., Nygaard and Pisoni, 1998) and that content from learned voices is better encoded in or recalled from memory (e.g., Martin et al., 1989). Other studies (Brungart and Simpson, 2004; Brungart et al., 2001) have also shown that prior knowledge of the vocal characteristics of either the target talker or a distracting talker improves performance in speech intelligibility tasks. In all, prior knowledge that allows focusing of attention on who is going to speak, and where and when this is going to occur, enhances speech intelligibility.

There is a relationship between cognitive processes such as attention, and the pupillary response (Beatty, 1982). Increased cognitive task demands reliably induce a larger pupil dilation response, allowing task-evoked pupillary responses to be used as a reliable and valid measure of cognitive processing load (Just et al., 2003; Kahneman and Beatty, 1966). Consequently, the task-evoked pupillary response quantifies listening effort in auditory tasks (Hyönä et al., 1995; Kramer et al., 1997). Generally, when a task requires more processing load in the same time interval, mean pupil dilation is larger when the task is being performed (Granholm and Verney, 2004). Additionally, in this same time window one can measure both the peak pupil dilation, which is thought to represent the maximum processing load, and peak latency, which is associated with processing time (Zekveld et al., 2011). The mean and peak pupil dilation are measured relative to a baseline, typically defined by the mean pupil diameter during a period of time in which no task-related processing occurs (e.g., over a time window one second prior to the onset of the target stimulus). In the current study, we analyzed all of these pupil measures, as they provide insight into how attention affects overall cognitive load (mean pupil dilation), maximum cognitive load (peak pupil dilation), processing speed of higher cognitive processes (peak latency), and overall task engagement (baseline), as explained below.

Pupil diameter is tightly linked to the activity of the Locus Coeruleus (LC) (Aston-Jones and Cohen, 2005; Gilzenrat et al., 2010). The noradrenergic system of the LC (LC-NE) is associated with various psychological processes, including attention. The activity of the LC-NE seems to exhibit two modes of function: phasic and tonic. During task performance, the phasic mode is associated with large responses to task-related events and low baseline firing rate of the LC-NE. The tonic mode is associated with high baseline activity of the LC-NE and a lack of phasic responses. The adaptive gain theory of Aston-Jones and Cohen (2005) proposes that the phasic mode is driven by optimization of performance (exploitation) and task engagement, whereas the tonic mode favors exploration of the environment, greater distractibility (sensitivity to task-irrelevant stimuli), and task disengagement. Rajkowski et al. (1994) investigated the relationship between the baseline pupil diameter and the LC-NE mode. The phasic and tonic modes were marked by relatively small and large baseline diameter values, respectively. It has been suggested that the task-evoked pupillary response corresponds to the phasic activity of the LC-NE, whereas the baseline pupil diameter corresponds to the tonic activity (Gilzenrat et al., 2010; Meer et al., 2010).

The main aim of the current study was to examine whether or not location, speech onset, and target talker uncertainty have an effect on the pupil response during speech perception tasks. Target location, onset, and talker variability were manipulated in three separate experiments. During these experiments, participants with normal hearing were presented with auditory sentences in fluctuating noise. Participants were asked to focus attention and repeat back target sentences while simultaneously ignoring distracting stimuli. We tested the hypothesis that allowing attention to focus on location, onset, or talker voice, would make it easier for listeners to filter out irrelevant information during early processing. Consequently, processing load would be reduced, as reflected by a smaller pupil dilation response and increased performance. In addition, participants gave subjective effort ratings after each task to allow us to evaluate how well cognitive load (specifically that related to attentional processes) reflects subjective listening effort.

## 2. Experiment 1: location uncertainty

In Experiment 1, we investigated the effect of location uncertainty on speech intelligibility and the pupil response (dilation, latency, baseline). We used a design similar to that employed in a previous study that examined the effect of divided attention on cognitive load (Koelewijn et al., 2014). In the current experiment, the location of the target speech was either fixed (location-fixed) during a block, by presenting sentences to the same ear, or varied (location-random) across trials, by randomly presenting the sentence to the left or right ear. We hypothesized that in the location-fixed condition participants would be able to focus spatial attention effectively, resulting in better speech intelligibility and a smaller pupil dilation response than for the location-random condition.

### 2.1. Methods

#### 2.1.1. Participants

Twenty-four young adults (age between 18 and 28 years, mean age 21.5 years) were recruited at the VU University Medical Center. All participants had normal hearing, defined as thresholds less than or equal to 20 dB HL over the frequency range from 0.25 to 4 kHz for both ears. All participants reported normal or corrected-to-normal vision, had no history of neurological disease, were native Dutch speakers, and provided written informed consent in accordance with the Ethics Committee of the VU University Medical Center.

#### 2.1.2. Task and materials

Two different everyday Dutch sentences obtained from a large set (Versfeld et al., 2000) were presented simultaneously via headphones, one to each ear. Each was masked by fluctuating noise (independent at the two ears). One sentence was spoken by a female talker and the other by a male talker. The presentation side of each talker was either randomized between trials or fixed during an entire block. Participants were informed about what condition was going to be presented before the start of each block. There were three tasks. In the ‘single-target’ task, participants were asked to report the sentence spoken by the female talker and ignore the sentence spoken by the male talker. In the ‘dual-target’ task, participants were instructed to first report the sentence spoken by the female talker (S1) and then report the sentence spoken by the male talker (S2). The stimuli were identical for the two tasks. In the control task, only one sentence, spoken by the female talker, was presented to one ear and participants had to report that sentence.

Each sentence had a level of 55 dB SPL. For each trial, randomly selected independent samples of fluctuating noise were added to the stimuli presented to each ear (in the control task, there was independent noise in the two ears, but no male talker in either ear). These samples were selected from a 5-min sound file. The fluctuating noise modulations mimicked the intensity fluctuations of speech of a single male talker (Versfeld et al., 2000) for two frequency bands, below and above 1 kHz (Festen and Plomp, 1990). The long-term average spectra of the fluctuating noise and sentences spoken by the male talker were matched to that of the sentences spoken by the female talker. The SNR was fixed at −9 dB, −3 dB, or +3 dB (see below) by changing the level of the fluctuating noise. This SNR range was chosen so that the single- and dual-target tasks as well as the control task would have average speech intelligibility levels above 50% words correct while staying below ceiling (Koelewijn et al., 2014). Within this range, the pupil dilation response has shown to be sensitive to the effects of masker manipulations independent of intelligibility or SNR (Koelewijn et al., 2012). The onset of the fluctuating noise was 3 s prior to the onset of both sentences and continued for 3 s after the end of the longer of the two sentences. The mean duration of the sentences was 1.9 s for the female talker (range = 1.3–2.7 s, SD = 0.26 s) and 2.0 s for the male talker (range = 1.3–2.9 s, SD = 0.30 s). At the end of each trial, a 1000-Hz prompt tone was presented for 1 s, after which participants were allowed to respond. Participants responded verbally and their response was scored in real time by the experimenter.

Participants were instructed to repeat back as many words as they could recall. The proportion of words correct per sentence was used as a performance measure. Presentations were blocked, with performance in each block corresponding to one of the three tasks. In each block, 10 trials per SNR were presented in random order, resulting in 30 trials per block. Note that the number of trials per SNR was lower than in our previous study (Koelewijn et al., 2014) in order to allow for the additional manipulation of location uncertainty while ensuring that the test session would not be so long as to induce fatigue. The six blocks (i.e., three tasks by two target-location conditions) were presented in an alternating order that was balanced over participants. Six sets of sentences were used; these were balanced between participants over blocks in a Latin square design to ensure that the order of sentences or combination of sentence and block (condition) did not confound the results. Prior to the experiment, participants were familiarized with the task by listening and responding to 6 practice trials for each condition (the order of these practice trials was also balanced over participants). After each block, participants were asked to indicate how well they thought they had performed the task, how much effort it took, and how motivated they were to perform the task, on a rating scale from 0 to 10. The whole procedure, including measurement of pure-tone hearing thresholds, practicing, fitting the eye-tracker, and performing the actual experiment with a 15-min break halfway through took approximately 2 h.

#### 2.1.3. Apparatus and procedure

Participants were seated in a sound-treated room at approximately 3.5-m viewing distance from a white wall. While listening to the sentences they had to fixate their gaze on a dot (diameter 0.47^−^) that was located at a height of 125 cm on the horizontal middle of the wall. An overhead light source illuminating the wall was placed at 3.5-m distance from the wall, outside the participants’ field of view. The light intensity was adjusted such that, for each participant, the pupil diameter was around the middle of its dynamic dilation range as measured by examination of the pupil size at 0 lx and 250 lx. During the task, the pupil diameter of the left eye was measured by an infrared eye-tracker (SMI, 2D Video-Oculography, version 4) with a spatial resolution of 33 pixels per centimeter and at a 50-Hz sampling rate. Separate files (44.1 Hz, 16 bit) for target sentences and maskers were mixed and presented binaurally from a PC by an external soundcard (Creative SoundBlaster, 24 bit, with optional processing turned off) through headphones (Sennheiser, HD 280, 64 Ω) by a dedicated program (written in MATLAB 2012a).

#### 2.1.4. Pupil data

**Fig. 1:**
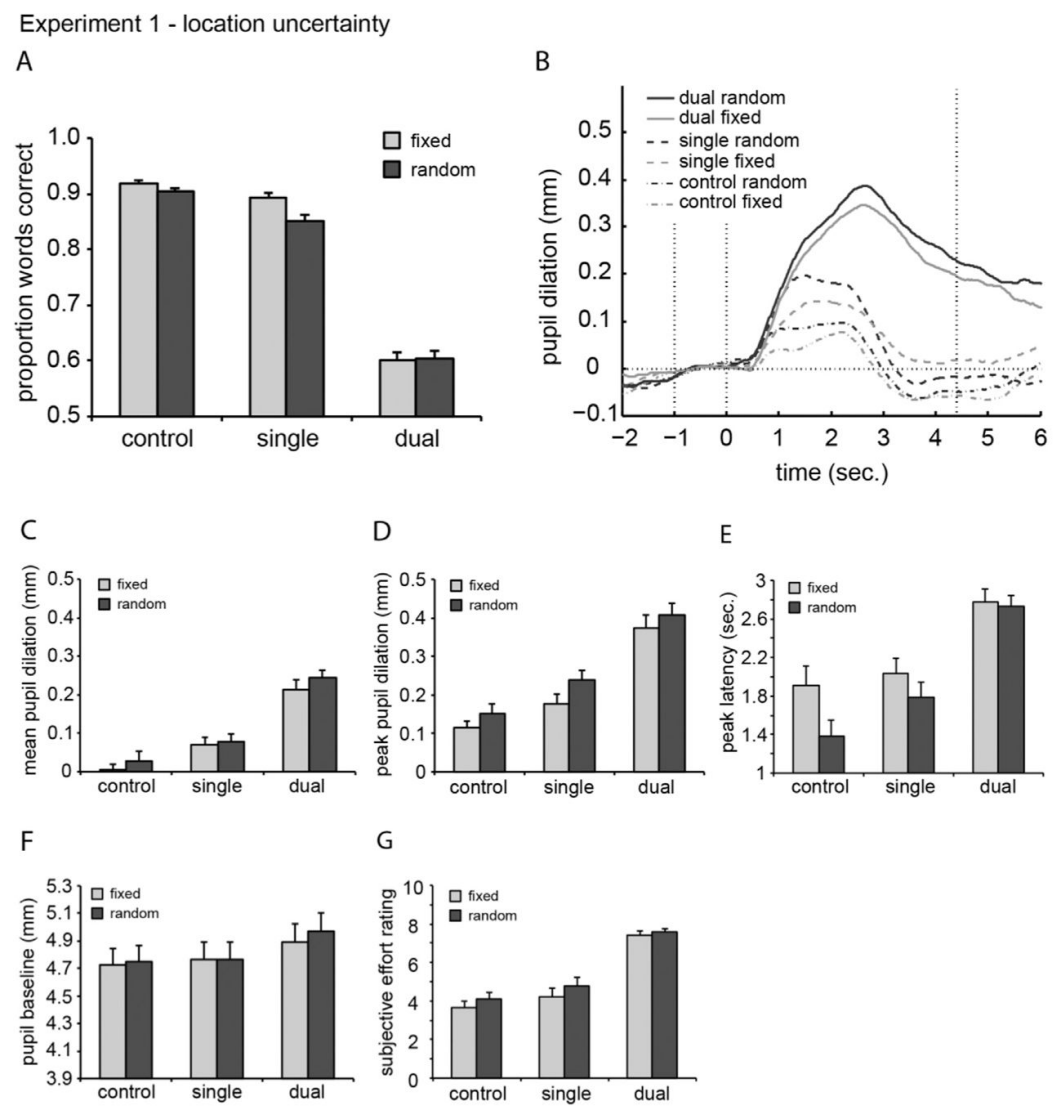
(A) Performance for each condition in Experiment 1, averaged over SNR and participants. Error bars indicate the standard error of the mean. (B) Pupil responses for each condition, averaged over SNR and participants. The onset of the sentences was at 0 s. The baseline, calculated as the average pupil diameter over one second preceding the start of the sentence, is shown by the dashed horizontal line. The time window over which the mean pupil dilation was computed corresponds to the range between the second and third dotted vertical lines. (C, D, E, F, and G) Pupil measures and subjective effort ratings for each condition, averaged over SNR and participants. Error bars indicate the standard error of the mean.

For each participant, the mean and SD of the pupil diameter were calculated for each pupil trace, recorded during each trial over a time period starting one second before sentence onset and ending at the start of the response prompt for the sentence with the shortest presentation time. Zero values and diameter values more then 3 SDs smaller than the mean diameter were coded as blinks. Traces in which more than 15% of their duration consisted of blinks were excluded from further analysis. For the remaining traces, blinks were removed by linear interpolation that started four samples before and ended eight samples after the blinks. The x- and y-coordinate traces of the pupil center (reflecting eye movements) were “deblinked” by application of the same procedure. A five-point moving average smoothing filter was passed over the deblinked pupil traces to remove any high-frequency artifacts. A spike detection algorithm was used to detect eye movements on both the x- and y-traces. This algorithm used a 100-ms time window sliding in 20-ms steps, in which the maximum amplitude differences were calculated between all possible time point combinations within the window. The SD was calculated for each x- and y-trace between the start of the baseline and the response prompt. All trials for which the maximum x- or y-amplitude difference exceeded 2 SDs were excluded from analysis. All remaining traces were baseline corrected by subtracting the trial’s baseline value from the value for each time point within that trace. This baseline value was the mean pupil size within the 1-s period prior to the onset of the sentence (when listening to noise alone), shown by the left and middle dotted vertical lines in Fig. 1B. Average traces were calculated separately for each participant and each condition. Within the average trace, mean pupil dilation was defined as the average pupil dilation relative to baseline within a time window ranging from the start of the sentence to the start of the response prompt, shown by the middle and right dotted vertical lines in Fig. 1B. Within this same time window, the peak pupil dilation was defined as the largest value relative to the baseline. The latency of the peak pupil dilation (ms) was defined relative to the sentence onset. Finally, for each participant and each condition the average baseline was calculated.

### 2.2. Results

#### 2.2.1. Performance data

The proportion of words correctly repeated was averaged over SNRs for the location-fixed and location-random conditions for the control, single, and dual-target tasks (Fig. 1A). For the conditions in the dual-target task, we computed the number of correctly reported words across both sentences divided by the total number of words presented in these sentences. Table 1 shows these scores, together with the proportion of correctly repeated words for the individual sentences in the dual-target task, and scores for the other conditions.

A two-way ANOVA on the *performance data* showed main effects of task (*F*_[2,46]_ = 452.44, *p* < 0.001) and location uncertainty (*F*_[1,23]_ = 8.07, *p* < 0.01). Additionally, there was an interaction between task and location uncertainty (*F*_[2,46]_ = 4.63, *p* = 0.015). Post-hoc analysis using three Bonferroni corrected two-sided paired-samples *t*-tests showed a significant difference between the location-fixed and location-random conditions for the single-target task (*p* < 0.01), but not for the control or dual-target task.

#### 2.2.2. Pupil data

Pupil traces containing a large number of eye blinks (2.2% of all trials) and/or large eye movements (9.0%) were removed from further analysis. For the remaining traces, the across-participant average peak pupil dilation, peak latency, and baseline values are presented in Table 1. In order to avoid effects of fatigue on the pupil dilation response, pupillometry experiments should have a limited duration. Therefore, the number of trials was restricted to 10 per SNR. After removal of the trials with too many blinks and/or eye moments, the number of trials that remained available for the analyses with SNR as additional independent factor was relatively small. We did not collect sufficient data for each participant to make a single analysis (i.e. with independent factors: task, condition, and SRN) feasible. Such an analysis would likely have been more sensitive to the effects of interest. Therefore, we proceeded with two separate ANOVAs: in one, we averaged pupil data over the tasks and conditions, and in the other, we averaged over SNRs. Note that by averaging the data across factor levels that may have influenced the data, we increase the variance that is unaccounted for in the ANOVAs, which made our analyses relatively conservative. First, to assess whether the current data replicated the effect of SNR on the mean pupil dilation observed by Koelewijn et al. (2014), we performed an ANOVA with SNR as independent factor, and pupil response (averaged over tasks and conditions) as dependent factor. The outcomes showed a significant effect of SNR (*F*_[2,46]_ = 19.60, *p* < 0.001). More negative SNRs resulted in a larger mean pupil dilation response, which is consistent with our previous results. Second, to examine the effect for each condition and task, we calculated the average pupil traces averaged over SNRs and across participants. The values are plotted in Fig. 1B. A two-way ANOVA on the *mean pupil dilation* data (Fig. 1C) showed a significant effect of task (*F*_[2,46]_ = 96.25, *p* < 0.001). The effect of location did not reach significance (*F*_[1,23]_ = 4.19, *p* = 0.052). No interaction between task and location uncertainty was observed (*F* < 1). A two-way ANOVA on the *peak pupil dilation* data (Fig. 1D), showed significant effects of both task (*F*_[2,46]_ = 69.94, *p* < 0.001) and location uncertainty (*F*_[1,23]_ = 9.81, *p* < 0.01). No interaction between task and location uncertainty was observed (*F* < 1). These results show that location uncertainty results in a larger pupil dilation response, which can be interpreted as more cognitive load.

A two-way ANOVA on *peak latency* (Fig. 1F) revealed a main effect of task (*F*_[2,46]_ = 29.72, *p* < 0.001) and location uncertainty (*F*_[1,23]_ = 6.90, *p* = 0.015) with a shorter latency in the random condition. Again, no interaction (*p* = 0.25) was observed. A two-way ANOVA on the *pupil baseline* data showed a main effect of task (*F*_[2,46]_ = 3.40, *p* = 0.042) but no effect of location uncertainty (*p* = 0.19) or interaction between task and location uncertainty (*F* < 1).

#### 2.2.3. Subjective data

The subjective effort ratings of one participant were excluded from analysis because of an incomplete form. A two-way ANOVA on *subjective effort* (Fig. 1G) revealed main effects of task (*F*_[2,44]_ = 65.78, *p* < 0.001), and location uncertainty (*F*_[1,22]_ = 9.91, *p* < 0.01), and no interaction (*F* < 1). These subjective effort effects are consistent with the pupil dilation data.

### 2.3. Discussion

Location uncertainty had an effect on both performance and the pupil peak dilation. Consistent with our previous results, performance decreased significantly when two sentences needed to be reported instead of one (Koelewijn et al., 2014). Consistent with other studies, performance was lower when the location of the target speech was uncertain (Kidd et al., 2005; Kitterick et al., 2010). However, this effect was only significant in the single-target condition and not in the dual-target condition, where both sentences had to be processed. Additionally, in the control condition, where there was no distracting sentence at the other ear, no effect of location uncertainly was observed, consistent with previous research (Kitterick et al., 2010). During the dual-target task, location information might have been irrelevant, since both sentences needed to be processed and neither sentence could be filtered out by an early attentional process. The fact that the control task showed no effect of location uncertainty may be due to a ceiling effect. Alternatively, since no distractor sentence needed to be filtered out, there may be no effect of location uncertainty in this condition.

The peak pupil dilation was lower in the fixed location condition, indicating reduced cognitive load. This objective measure of effort agreed with subjective performance and effort scores, which both showed an effect of task and location uncertainty. Also, peak pupil dilation was larger and peak latency shorter in the location-random condition than in the location-fixed condition. This could indicate that more cognitive resources were used in the location-random condition than in the location-fixed condition. These additional resources could in turn decrease the actual speech processing time. In contrast, in the dual task, additional resources are likely needed to process the increased information in the two sentences, which may explain why the latency was not reduced compared to the single task. Finally, the pupil baseline seemed to increase with task difficulty. A similar effect was shown in our previous study (Koelewijn et al., 2014), and was explained as an anticipated task difficulty effect.

## 3. Experiment 2: onset uncertainty

In Experiment 2, we assessed the effect of speech onset uncertainty on speech intelligibility and the pupil response. We used the same design as in Experiment 1, with the exception that only the single-target task was used. We hypothesized that when speech onset time was constant (onset-fixed) and predictable, participants could use this as an implicit temporal cue. This would allow them to focus attention on a specific time window spanning the duration of the sentence. This should result in better speech intelligibility and a smaller pupil dilation response compared to when the target timing was random (onset-random).

### 3.1. Methods

Sixteen young adults (age between 19 and 33 years, mean age 26.3 years) recruited at the VU University Medical Center were included. All participants had normal hearing, normal or corrected-to-normal vision, no history of neurological diseases, and were native Dutch speakers. All provided written informed consent.

In the single-target task, the target sentence was always uttered by the same female talker and always presented to the left ear against a background of fluctuating noise. The distractor sentence (in noise) was always uttered by the same male talker and presented to the right ear. The onset times of the target and distractor sentences were manipulated. In the onset-fixed condition, within a block, both sentences began 4 s after the onset of the fluctuating noise. In the onset-random condition, the two sentences were still simultaneously presented, but started randomly at 2, 4, or 6 s after the noise onset. The rationale for this approach was that participants would implicitly know the onset in the onset-fixed condition, but not in the onset-random condition. To make sure that the speech onset in the 6-s trials was unpredictable despite the fact that after 4 s, participants could exclude the possibility of onsets at 2 or 4 s, in 25% of the trials no sentences were presented and the participants heard only noise. To make sure that the only thing changing between blocks was onset uncertainty and not the probability of a sentence occurring, 25% of the trials in the fixed-onset condition also contained no sentences. Each block contained 12 trials for each SNR and 12 trials in which no sentences were presented, for a total 48 trials. The order of the trials was randomized within each block, and the order of the blocks was balanced over participants. All other procedures and equipment were identical to those used in Experiment 1. The whole experiment took approximately 1 h per participant.

### 3.2. Results

#### 3.2.1. Performance data

**Fig. 2:**
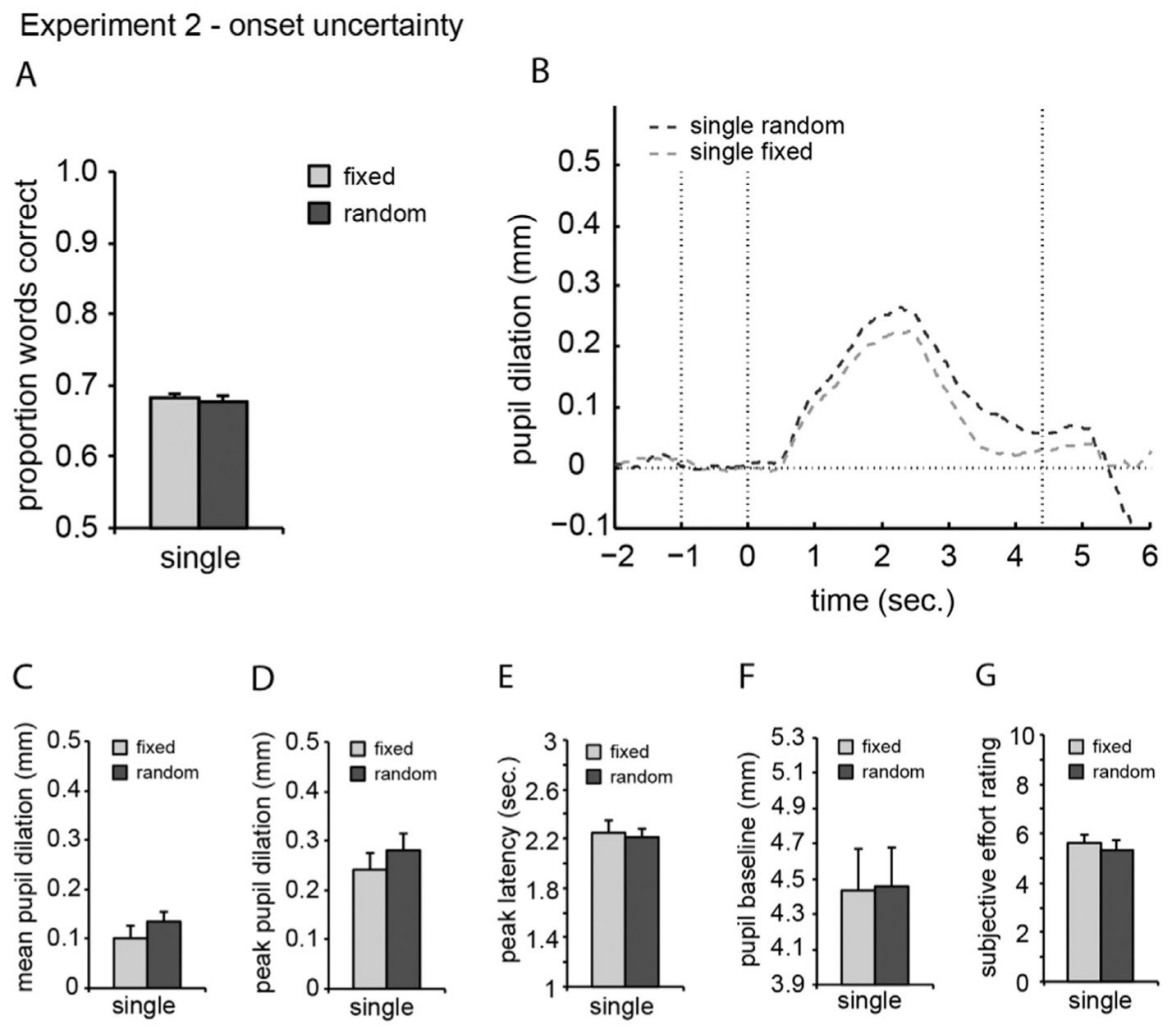
As Fig. 1, but for Experiment 2.

The proportion of words correct averaged over SNRs is shown for each condition in Fig. 2A. Table 2 shows the proportion of words correct averaged over participants. Paired-samples *t*-test showed no significant difference in *performance* between the fixed and random conditions (*p* = 0.53).

#### 3.2.2. Pupil data

Pupil traces containing a large number of eye blinks (4.4%) and/ or large eye movements (5.6%) were removed from further analysis. Traces for the trials containing no sentences were also excluded from analysis. For the remaining traces, the across-participants average mean and peak pupil dilation, peak latency, and baseline values are presented in Table 2. The average pupil traces for each condition and task, averaged over SNRs and across participants, are plotted in Fig. 2B. Paired-samples *t*-tests were performed comparing the *mean pupil dilation* (*p* = 0.13), *peak pupil dilation* (*p* = 0.19), *peak latency* (*p* = 0.77), and *baseline* (*p* = 0.46) for the fixed and random conditions. There were no significant effects.

#### 3.2.3. Subjective data

There was no significant difference in subjective effort between the fixed and random conditions.

### 3.3. Discussion

Uncertainty in the onset time of the target speech did not affect performance or the pupil dilation response. Additionally, subjective scores were similar for onset-fixed and onset-random conditions. These results differ from previously observed effects of target timing uncertainty on performance (Kitterick et al., 2010). This difference might be explained by less onset uncertainty in the current study compared to the study of Kitterick and colleagues.

## 4. Experiment 3: talker uncertainty

Vocal characteristics, including pitch, timing, and timbre, differ across talkers (Brungart, 2001; Kraus and Chandrasekaran, 2010). These differences can be used to orient one’s attentional focus onto target speech and filter out (ignore) the voices of other distracting talkers. Several studies suggest that talker/voice uncertainty affects the ability to understand a target talker amidst other talkers (Brungart et al., 2001; Brungart and Simpson, 2004; Kitterick et al., 2010). To date, however, very little is known about how this factor affects listening effort.

In Experiment 3, we determined the effect of target talker uncertainty on the pupillary response and task performance while a distractor sentence was either present (the single-target task) or absent (in the control task), based on previous results (Kitterick et al., 2010) showing that talker uncertainty has a greater effect on performance when there is a distractor sentence.

### 4.1. Methods

Sixteen normal-hearing participants between the ages of 19 and 33 years (mean age of 25.4 years) were recruited at the VU University Medical Center. During the course of data collection two participants were replaced because of unreliable eye tracking. All participants met the same criteria as before and all provided written-informed consent.

In the single-target task, participants listened through headphones to two Dutch sentences, one in each ear, while in the control task only one sentence was presented to one ear. In both tasks, independent samples of fluctuating noise were presented to both ears. Although the structure and content of the sentences were similar to those used in Experiments 1 and 2, this time the sentences were gathered from the other two out of the four sets described by Versfeld et al. (2000). Each set contains the same sentences uttered by four different talkers: two male (coded as AM and RB) and two female (coded as HB and MS). From each set, the same 384 sentences were selected, based on intelligibility, articulation, and whether every talker uttered all words in the correct order. The audio files of these uttered sentences differed in length. The mean duration was 1.9 s for talker AM (range = 1.4–2.9 s, SD = 0.2), 1.8 s for talker HB (range = 1.3–2.6 s, SD = 0.2), 1.8 s for talker MS (range = 1.3–3.0 s, SD = 0.2), and 2.0 s for talker RB (range = 1.3–3.1 s, SD = 0.3). To equate the amount of energetic masking of all four talkers by the fluctuating noise, the power spectrum for each talker was adjusted to match the mean power spectrum. Additionally, the power spectrum of the fluctuating noise was adjusted to have this power spectrum.

The target sentence was always presented to the left ear and was either uttered by the same talker during the entire block (talker-fixed condition) or randomly selected from one of the four talkers (talker-random condition). In the single-target task, the distractor sentence presented at the right ear was randomly selected from one of the other three talkers. The presentation order of the four blocks (two tasks by two talker-uncertainty conditions), each containing 36 trials (12 for each SNR), was balanced over participants. Furthermore, across participants, each of the four talkers was used as the target an equal number of times. Other methods were identical to those used in Experiment 1. The whole procedure took approximately 2 h per participant.

### 4.2. Results

#### 4.2.1. Performance data

**Fig. 3:**
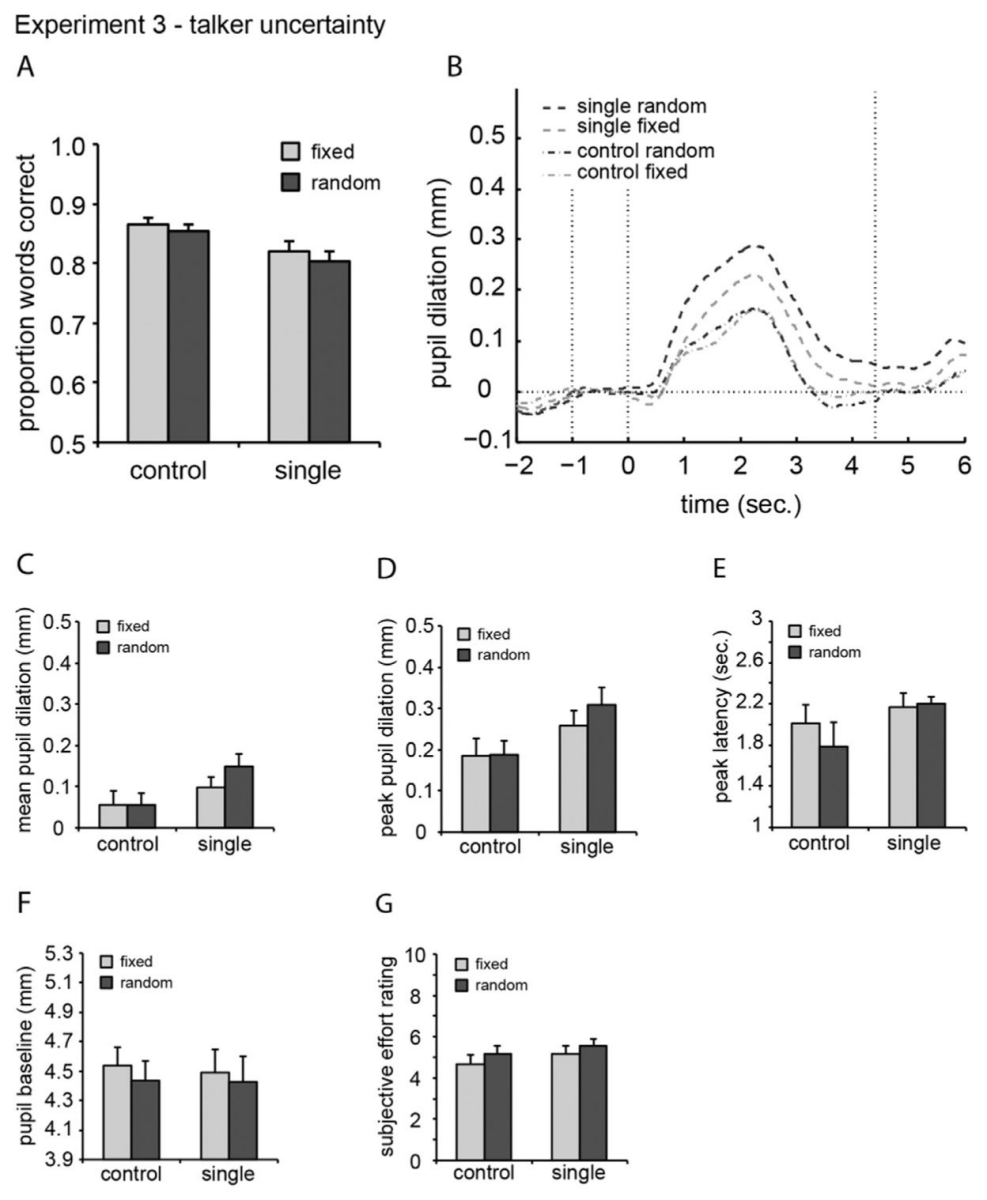
As Fig. 1, but for Experiment 3.

The proportion of words correct averaged over SNRs for both tasks and the talker-fixed and talker-random conditions, is shown in Fig. 3A. The proportion of words correct averaged over participants is shown in Table 3. A two-way ANOVA on the *performance data* showed a significant effect of task (F[1,15] = 15.286, *p* < 0.001), no effect of target talker uncertainty (F[1,15] 1.536, *p* = 0.234) and no interaction (F < 1). Participants performed better when no distracting talker was present.

#### 4.2.2. Pupil data

Pupil traces containing a large number of eye blinks (4.4%) and/ or large eye movements (7.9%) were removed from further analysis. For the remaining traces, the across-participant average mean and peak pupil dilation, peak latency, and baseline values are presented in Table 3. The average pupil traces for each condition and task, averaged over SNRs and across participants, are plotted in Fig. 3B. A two-way ANOVA on the *mean pupil dilation* data (Fig. 3C) showed a significant effect of task (F[1,15] = 11.256, *p* < 0.01), no effect of talker uncertainty (F[1,15] = 2.594, *p* = 0.128), and no interaction (F [1,15] = 2.752, *p* = 0.118). A two-way ANOVA on the *peak pupil dilation* data (Fig. 3D) showed a significant effect of task (*F*_[1,15]_ = 14.63, *p* < 0.01), no effect of talker uncertainty (*F*_[1,15]_ = 1.79, *p* = 0.200), and no interaction (*F*_[1,15]_ = 1.22, *p* = 0.286).

A two-way ANOVA on *peak latency* (Fig. 3E) revealed no effect of task (*F*_[1,15]_ = 2.60, *p* < 0.128), no effect of talker uncertainty (F < 1), and no interaction (F < 1). A two-way ANOVA on *pupil baseline* (Fig. 3F) showed no effect of task (F < 1) or interaction between task and talker uncertainty (F < 1). Interestingly, there was a main effect of talker uncertainty (*F*_[1,15]_ = 4.99, *p* < 0.041); baseline was smaller for the talker-random condition than for the talker-fixed condition.

#### 4.2.3. Subjective data

The subjective effort ratings of one participant were excluded from analysis because of an incomplete form. A two-way ANOVA on *subjective effort* (Fig. 3G) showed a significant effect of talker uncertainty (*F*_[1,14]_ = 2.46, *p* = 0.047). No task effect or interaction was observed.

### 4.3. Discussion

Target-talker uncertainty did not influence the task-evoked pupil dilation. However, in the talker-uncertain condition, the difference between random versus fixed in the single-target task in Fig. 3B was similar to the effect size of location uncertainty in the single-target task in Experiment 1 (Fig. 1B). Therefore, we performed two-sided paired t-tests on the mean and peak pupil dilation. These showed a significant difference in mean pupil dilation between the talker-fixed and talker-random conditions in the single-target task (*p* = 0.036) but not in the control task (*p* = 0.956). Note that this effect did not lead to a significant main or interaction effects and, therefore, should be interpreted with caution. There was no significant difference in peak pupil dilation between the talker-fixed and talker-random conditions in either the single-target task (*p* = 0.140) or the control task (*p* = 0.952). The mean pupil dilation and peak pupil dilation were significantly larger during the single-target task than during the control task. These findings, together with earlier results (Koelewijn et al., 2014, 2012; Zekveld and Kramer, 2014), suggest that cognitive processing load is greater when a distractor sentence is present. The baseline pupil diameter was significantly lower when there was no prior knowledge of the target talker identity. No differences in baseline pupil diameter were seen between tasks. Surprisingly, unlike in Kitterick et al. (2010), there was no significant effect of target-talker uncertainty on performance. This suggests that target-talker uncertainty may influence pupillary response parameters even when performance is not affected. Alternatively, the lack of effect may be due to a difference in power between the work of Kitterick and colleagues and the current study, compounded by the fact that the unpredictability that subjects faced in their study was greater than that tested here, potentially enabling them to observe significant effects where we did not.

## 5. General discussion

The results showed effects of target location on cognitive processing load using the task-evoked pupillary response. When location was fixed rather than random, the pupil dilation response was smaller. Furthermore, when the talker was fixed the pupil baseline was larger than when the talker was random. In Experiments 1 and 3, participants performed better and the pupil dilation response was smaller when no distracting sentence was presented than when a distractor sentence was presented. In Experiment 1, performance dropped and the pupil dilation response increased when participants had to recall two as opposed to one sentence. These results are consistent with previously shown effects of informational masking (Koelewijn et al., 2012) and divided attention (Koelewijn et al., 2014) on task performance and cognitive load. Interestingly, the effects of attention on the pupil dilation response and the subjective listening-effort ratings were consistent. This suggests that the deployment of attention during speech processing in adverse listening conditions affects listening effort.

In Experiment 2 we did not find a significant effect of speech onset uncertainty on performance or on the pupil response. As mentioned in the introduction, previous studies observed small effects of temporal cues on performance (Akeroyd, 2008; Best et al., 2007; Kitterick et al., 2010). Although Kitterick et al. (2010) did find an effect on performance, their experimental space was far more complex, containing multiple locations and talkers, and unpredictable sentence timing. This creates a situation with many potential target and distractor onsets, which makes actual information about target-speech onset more relevant. Although the current study tried to establish the relevance of temporal features in isolation, it may be that effects of temporal uncertainty are modest unless listeners are in a more complex listening environment; only then might knowledge about when someone is going to speak prove important for deploying resources. Therefore, the current negative results are inconclusive about whether speech onset uncertainty affects listening effort.

Remarkably, performance in Experiment 3 was not affected by target talker uncertainty. In contrast, Kitterick et al. (2010) showed that participants were able to perform an auditory task in a multi-talker environment with lower SNRs when the target talker uncertainty was less. Differences in experimental design across the two studies may account for the different outcomes. First, the study of Kitterick et al. (2010) simultaneously addressed the effects of location and speech-onset cues, while the current study investigated these effects in isolation. Second, Kitterick et al. (2010) used at least twelve distracting phrases within each trial instead of the one used in the current study. Third, in the current study one of four talkers could utter the target sentences while Kitterick et al. (2010) used one of eight talkers. It may be that prior knowledge of the target talker is more beneficial when the number of possible target talkers (or talker uncertainty) is greater. As opposed to Kitterick et al. (2010), here speech was masked by fluctuating noise rather than competing speech streams. The audibility of speech might affect how well we perceive differences between voices, which could alter the effect of the talker uncertainty manipulation. Finally, a negative result cannot be used to conclude that there is no effect of the manipulations tested in the current experiment; it may be that the effects were too small to lead to significant differences in the current study, especially given the differences in experimental design and task complexity.

For talker uncertainty, the baseline pupil size was significantly larger when the same speaker uttered the target sentences than when the target talker varied. According to the adaptive gain theory of Aston-Jones and Cohen (2005), the baseline pupil diameter may be associated with participants’ engagement in the task. According to this theory, the phasic mode reflects task engagement and is characterized by a small baseline while the tonic mode reflects exploration and is associated with a large baseline. Remarkably, this is not consistent with the observed talker uncertainty effect in the current study, where the fixed (engaged) condition showed a larger baseline than the random (exploratory) condition. One could argue that the random condition was the more difficult condition and therefore needed more task engagement, resulting in a smaller pupil baseline. However, this is not backed up by the performance data of Experiment 3. Thus, the baseline data of Experiment 3 cannot be explained by the adaptive gain theory.

An alternative explanation for how the pupil baseline is affected by attention is that there is relationship between the pupil baseline and the pupil dilation response. Remember that the results of Experiment 1 showed a larger baseline for the dual-target task than for both other tasks. We proposed previously that this was related to task difficulty (Koelewijn et al., 2014). This task effect suggests that more cognitive resources are recruited for more complex tasks, and that this is reflected by a higher baseline. When these resources in turn are set to work during speech processing, this should lead to a larger pupil dilation response, which indeed was the case in Experiment 1. This suggests that the pupil baseline and the pupil dilation responses are inter-related.

In Experiment 1, peak latency was shorter in the location-random than in the location-fixed condition. As suggested in the discussion of Experiment 1, it could be that more cognitive resources were used during the location-random condition. The reason why more cognitive resources were available could be related to what was observed in the baseline data of Experiment 3. We speculate that if resources are needed in order to focus attention in the fixed condition, as the pupil baseline data suggest, then in the *random* condition there are more resources available to process the incoming information. As suggested earlier, the availability of more resources could speed up processing, resulting in shorter peak latencies. In all, there seems to be a correspondence between the cognitive resources available at a certain time and how much of the resources is used, as reflected by the pupil baseline, peak latency, and pupil dilation response.

Last but not least, we showed that the subjective scores were consistent with the pupil dilation responses. Both location and talker uncertainty had an effect on the pupil dilation response that was consistent with the subjective effort ratings. This is in addition to the task effect shown in Experiment 1 that was similarly reflected in pupil scores and effort ratings (also consistent with Koelewijn et al., 2014). Pupil dilation and subjective listening effort are more similar in the way they respond to manipulations of attention than what was observed previously for effects like intelligibility level and informational masking (e.g., Koelewijn et al., 2012).

## 6. Conclusions

The current results show that, in a cocktail party situation, listening effort increases when listeners are uncertain about the acoustic features that differentiate the target from the distractor, like location or the voice of the target talker. Although uncertainty about the speech timing did not affect the pupil dilation response, here it was studied in isolation; timing uncertainty may well interact with other features, as has been shown to be the case for performance in more complex, unpredictable conditions (e.g., Kitterick et al., 2010). Based on both the current and previous pupillometry data and subjective effort scores (Koelewijn et al., 2014), there is a strong indication that listening effort is closely tied to both the amount of attentional resources required and the efficiency and effectiveness with which these attentional resources can be deployed during speech processing in adverse listening conditions.

## Figures and Tables

**Table 1:** Results of Experiment 1, showing average performance scores, peak dilation values, and subjective effort scores as a function of location for each condition.

	Location	
	Fixed	Random
Performance	Proportion words correct (SD)	
Control	0.92 (0.04)	0.90 (0.03)
Single**	0.89 (0.05)	0.85 (0.06)
Dual	0.60 (0.08)	0.60 (0.08)
Pupil	Mean dilation (SD), mm	
Control	0.01 (0.07)	0.03 (0.12)
Single	0.07 (0.11)	0.08 (0.10)
Dual	0.21 (0.11)	0.24 (0.09)
	Peak dilation* (SD), mm	
Control	0.12 (0.09)	0.15 (0.12)
Single	0.18 (0.12)	0.24 (0.11)
Dual	0.37 (0.16)	0.41 (0.14)
	Peak latency* (SD), s	
Control	1.91 (1.00)	1.38 (0.86)
Single	2.03 (0.80)	1.78 (0.80)
Dual	2.78 (0.63)	2.73 (0.63)
	Baseline (SD), mm	
Control	4.73 (0.56)	4.75 (0.56)
Single	4.76 (0.62)	4.77 (0.62)
Dual	4.89 (0.66)	4.97 (0.68)
Subjective effort*	Scores (SD) (low = 0, high = 10)	
Control	3.63 (1.78)	4.10 (1.82)
Single	4.23 (2.12)	4.80 (2.19)
Dual	7.40 (1.27)	7.57 (1.01)

* Significant mean effect of location uncertainty in the absence of an interaction.

** Significant effect of location uncertainty when post-hoc analysis was performed to test an interaction.

**Table 2:** Results of Experiment 2, showing average performance scores, peak dilation values, and subjective effort scores as a function of onset for each condition.

	Time	
	Fixed	Random
Performance	Proportion words correct (SD)	
Single	0.68 (0.02)	0.68 (0.03)
Pupil	Mean dilation (SD), mm	
Single	0.10 (0.10)	0.13 (0.08)
	Peak dilation (SD), mm	
Single	0.24 (0.13)	0.28 (0.12)
	Peak latency (SD), s	
Single	2.24 (0.40)	2.21 (0.28)
	Baseline (SD), mm	
Single	4.43 (0.94)	4.46 (0.88)
Subjective effort	Scores (SD) (low = 0, high = 10)	
Single	5.60 (1.45)	5.34 (1.62)

**Table 3:** Results of Experiment 3, showing average performance scores, peak dilation values, and subjective effort scores as a function of location for each condition.

	Talker	
	Fixed	Random
Performance	Proportion words correct (SD)	
Control	0.86 (0.05)	0.85 (0.04)
Single	0.82 (0.06)	0.80 (0.06)
Pupil	Mean dilation (SD), mm	
Control	0.06 (0.13)	0.06 (0.12)
Single	0.10 (0.10)	0.15 (0.13)
	Peak dilation (SD), mm	
Control	0.19 (0.16)	0.19 (0.14)
Single	0.26 (0.15)	0.31 (0.17)
	Peak latency (SD), s	
Control	2.01 (0.72)	1.79 (0.94)
Single	2.17 (0.52)	2.20 (0.25)
	Baseline* (SD), mm	
Control	4.54 (0.52)	4.44 (0.54)
Single	4.49 (0.63)	4.42 (0.69)
Subjective effort*	Scores (SD) (low = 0, high = 10)	
Control	4.68 (1.66)	5.16 (1.54)
Single	5.17 (1.67)	5.55 (1.42)

* Significant mean effect of target talker uncertainty in the absence of an interaction.
